# Microfluidic Integration of Magnetically Functionalized Microwires for Flow Cytometry Protein Quantification

**DOI:** 10.3390/ma18020215

**Published:** 2025-01-07

**Authors:** Liviu Clime, Catalin Pavel, Lidija Malic, Christina Nassif, Matthias Geissler, Nicoleta Lupu, Tibor-Adrian Óvári, Lucas Poncelet, Gaétan Veilleux, Elham Moslemi, Javier Alejandro Hernández-Castro, Daniel Sinnett, Diping Che, Teodor Veres

**Affiliations:** 1 Life Sciences Division, National Research Council of Canada, 75 de Mortagne Boulevard, Boucherville, QC J4B 6Y4, Canadamatthias.geissler@cnrc-nrc.gc.ca (M.G.); lucas.poncelet@cnrc-nrc.gc.ca (L.P.); javier.hernandez-castro@cnrc-nrc.gc.ca (J.A.H.-C.);; 2 Azure Biosystems, 4810 Jean Talon O., Suite 328, Montreal, QC H4P 2N5, Canada; 3 National Institute of Research and Development for Technical Physics (NIRDTP), 47 Mangeron Boulevard, 700050 Iași, Romania; 4 Centre Hospitalier Universitaire Sainte-Justine, 3175 Chemin de la Côte-Sainte-Catherine, Montreal, QC H3T 1C5, Canada

**Keywords:** microfluidics, protein quantification, magnetic microwires, magnetic nanoparticles, nanocrystalline magnets, flow cytometry

## Abstract

A novel approach to protein quantification utilizing a microfluidic platform activated by a magnetic assembly of functionalized magnetic beads around soft magnetic capture centers is presented. Functionalized magnetic beads, known for their high surface area and facile manipulation under external magnetic fields, are injected inside microfluidic channels and immobilized magnetically on the surface of glass-coated soft magnetic microwires placed along the symmetry axis of these channels. A fluorescent (Cy5) immunomagnetic sandwich ELISA is then performed by sequentially flowing the sample and all necessary reagents in the microfluidic channels. Direct protein quantification is performed by magnetically releasing the beads from the microwire and evaluating their fluorescence intensity with the help of a miniature (microfluidic-based) flow cytometer. Measurements of ICAM-1 protein concentration in human blood plasma samples confirm the feasibility of the approach through extensive performance benchmarking. The automation and multiplexing capabilities of the proposed platform further demonstrate its potential for protein quantification in point-of-care settings using microfluidics and miniature flow cytometry instruments.

## 1. Introduction

Protein quantification is a critical process in biological research and clinical diagnostics as it plays a pivotal role in understanding cellular functions, elucidating disease mechanisms, and guiding therapeutic treatment. Techniques such as UV spectrophotometry [1], mass spectrometry [2], and enzyme-linked immunosorbent assay (ELISA) [3] are commonly employed to achieve accurate quantification. Recent advancements in microfluidic technologies have further enhanced protein quantification by enabling miniaturization, which reduces reagent consumption and allows for high-throughput analysis [4]. In addition, these systems offer tailored solutions for fluid delivery, manipulation, and dosage, up-stream sample preparation, target isolation and enrichment, as well as derivatization, binding, and labeling of assay components. As such, microfluidics can improve efficiency in analyzing complex biological samples. Engineering fluidic elements (such as reservoirs and channels) with high precision is key to harnessing the potential of these devices [5,6,7], while manufacturability, cost, and ease of manipulation are important considerations for widespread adoption [8].

Several limitations related to the costs of production along with other biocompatibility and chemical issues shifted the attention from traditional materials such as glass or silicon to polymers: they are relatively inexpensive and have a wide range of properties to choose from while offering good optical transparency and electrical insulation [9]. In addition to these advantages, thermoplastic polymers are compatible with high-volume production processes such as hot embossing, roller embossing or injection molding [9,10]. Additionally, some polymer materials—such as polycarbonate—are compatible with several fabrication processes, allowing for the combination of several fabrication techniques depending on the desired outcome. For instance, CNC (Computer Numerical Control) routers can be employed for low-volume production [11,12], usually aimed at research and optimization purposes, while injection molding or hot embossing are reserved to large-scale manufacturing and commercialization [13]. 

The biofunctional properties of the materials represent another crucial aspect of device manufacturing. Biofunctionalization strategies usually involve surface treatments such as hydroxylation, amination, carboxylation, biotinylation, etc., applied directly to the interior (i.e., the inner surfaces) of microchannels [6]. These treatments are generally time-consuming since they involve several chemical modification steps which increase costs and limit mass production and commercialization [6]. One alternative is to use magnetic beads due to their unique properties, including high surface area, facile functionalization in batch format, and ease of manipulation under an external magnetic field [14]. Moreover, magnetic particles enhance specificity and binding affinity, enabling precise capture and detection [15]. Several innovative microfluidic platforms leveraging these advantages have been developed, including electrically actuated digital microfluidic (EWOD) systems [16,17], arrays of pillars for magnetic bead capture [18], and magnetically actuated microposts [19]. They offer improved control over sample manipulation, enhanced capture efficiency, and compatibility with various assay formats [20], making it possible for diverse protein quantification methods to be integrated into microfluidic systems, including colorimetric detection with ELISA [21] or magnetic sensing based on the giant magnetoresistance (GMR) effect [22]. While providing high sensitivity and specificity, enabling accurate quantification of target proteins within complex biological samples, these approaches generally mimic standard manual assays in a microfluidic format, offering limited gains in terms of degree of automation, throughput, and overall assay time.

Here, we present an approach to the functionalization of microfluidic devices through the magnetic capture of functionalized magnetic beads on magnetic capture centers in the form of long metal wires placed inside and along microfluidic channels. The beads are functionalized in bulk and introduced in the microfluidic device as a first step in the testing procedure. The sample and reagents are subsequently pumped along the microfluidic channels by using programmable and automated pumping and distribution valves which are compatible with the pumping systems employed in flow cytometry instruments for seamless integration. Rapid quantification of the proteins (ICAM-1) captured on the beads is carried out by resuspending these beads in clean buffers and flowing them through a miniature microfluidic flow cytometer. We focus on ICAM-1 (Intercellular Adhesion Molecule 1) [23], which plays a role in leukocyte engagement during inflammation. We demonstrate a dynamic range from 0 to 40 ng/mL in human plasma samples with a precision of about 0.5 ng/mL and a maximum sensitivity at an optimal dilution factor of 1/10. Furthermore, the multiplexing capabilities of the proposed platform are highlighted, along with the potential for protein quantification using portable flow cytometers and point-of-care instruments.

## 2. Materials and Methods

**Figure 1:**
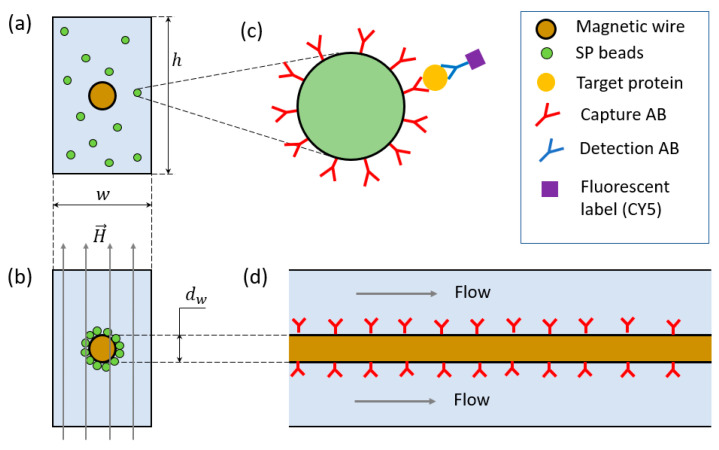
(**a**) Schematic drawing of a cross-sectional view of a microfluidic channel of width w and height h with the magnetic wire at the center filled with a colloidal suspension of functionalized magnetic beads floating freely in the solution. (**b**) Functionalization of the wire through magnetization in the transversal direction using an external magnetic field $\vec{H}$. (**c**) Schematic illustration of the sandwich ELISA implemented on the magnetic beads. (**d**) Longitudinal cross-sectional drawing of a section of the microfluidic channel highlighting the positioning of the wire and the direction of the flow.

The prototype instrument demonstrated here has been assembled by combining several commercially available parts (a syringe pump, a distribution valve, and a flow cytometry instrument) with in-house fabrication (microfluidic devices, world-to-chip interfacing) and automation. The main idea consists of implementing a complete sample-to-answer immunomagnetic assay by functionalizing a microfluidic chip on-the-fly through the immobilization of functionalized magnetic beads on magnetic capture centers inside microfluidic channels that serve as incubation reservoirs (Figure 1). The functionalized beads are introduced first in the microfluidic channel as a highly concentrated colloidal suspension ($10^{7}\;mL^{-1}$) and immobilized on the surface of a magnetic microwire (Figure 1a) positioned along the symmetry axis of the microfluidic channel (as described below in Section 2.1). To achieve a uniform coating and optimal capture efficiency, the applied external magnetic field must be both uniform and strong enough to magnetize the wire and the beads as close to saturation as possible. Once covered with functionalized magnetic particles (Figure 1b), the wire inside the microfluidic channel behaves as a functionalized surface, allowing the immunoassay to be conducted by following standard protocols where the sample and all necessary reagents flow through the microfluidic channels (Figure 1c,d). The immunoassay for capturing the target protein (here ICAM-1) is engineered so that, at the end of the microfluidic assay, the beads are labeled with a Cy5 fluorescent dye, with the amount of fluorescence on their surface being proportional to the number of captured proteins. A direct physical measurement of this quantity is then performed by removing the beads from the surface of the wire and transferring them to the flow cytometry instrument for fluorescence quantification (as described in Section 2.6).

### 2.1. Microfluidic Device

**Figure 2:**
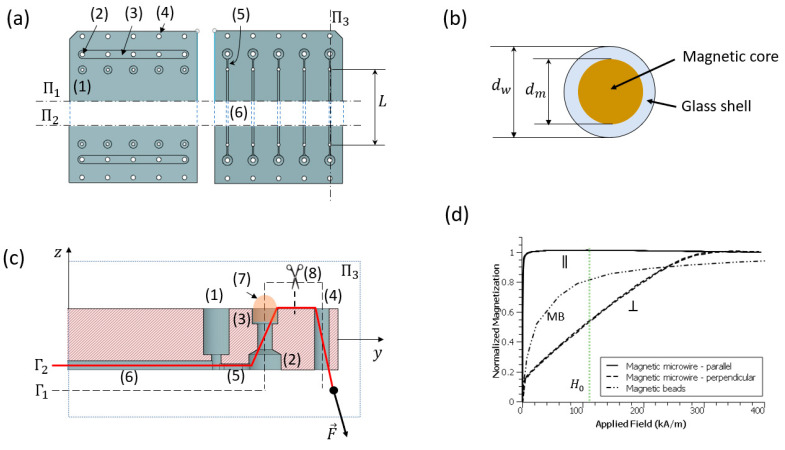
(**a**) Schematic drawings of the microfluidic device indicating the essential features of the design: the inlets and the outlets (1), the wire installation and tensioning holes (2) and (4), the wire fixing glue cavity (3), and the self-aligning channels (5). For figure footprint optimization purposes, the drawing excludes the central part of the device. (**b**) Cross-sectional view of the magnetic microwire used in this study featuring the magnetic core of dimeter $d_{m}=25\;\mu m$ covered by a glass shell of 15 μm thickness for a total diameter of the wire $d_{w}=55\;\mu m$. (**c**) Longitudinal vertical sectional view along one channel of the microfluidic device featuring the outlet (1) and the additional features (2–7) for installing, aligning and fixing the wire in place. (**d**) Magnetization curves for the wire on the parallel (∥) and perpendicular (⊥) directions for the wire and for the magnetic beads (MB).

The microfluidic devices are designed with Fusion 360 (Autodesk, San Francisco, CA, USA) and fabricated with a CNC milling machine (Q350, Menig Automation, Morgan Hill, CA, USA) from injection-molded cyclic olefin polymer (COP) blanks (Zeonor, mcs-COP-02, 6 mm in thickness; microfluidic ChipShop, Jena, Germany). The final assembled device is in the shape of a rectangle (2.5 cm×7.5 cm) featuring 5 microfluidic channels (400 μm width, 600 μm height, and 6 cm long) with inlet and outlet ports equally spaced near the short sides (labeled as (1) in Figure 2a). The fabrication process employed here is capable of reproducing microfluidic features with micrometric precision, with the most important source of errors originating from manually setting the zero level on the Z direction. Note that in Figure 2a we illustrate only the two regions containing the connecting ports, omitting the central section (between the cut planes $\Pi_{1}$ and $\Pi_{2}$), where the only present features are the 5 parallel microfluidic channels. In addition to these connection ports, several other features are included in order to allow proper aligning and installation of the magnetic microwire (National Institute for Research and Development in Technical Physics—NIRDTP, Iasi, Romania) inside the channels: the pairs of holes (2) and (4) at each channel end for applying tension on the wire, the grooves (2) for fixing the tension in the wire by filling it with UV glue (7), and the alignment channel (5) situated at the ends of the channels between the fluidic port (1) and the first wire tensioning hole (2), to precisely position the wire along the symmetry axis of each channel (6). The magnetic wire has a magnetically soft metallic core $d_{m}=25\;\mu m$ (Figure 2b) made of a FeCuNbSiB alloy and covered with a thin silica shell such that the total diameter is $d_{w}=55\;\mu m$ (more details are given in Section 2.2).

A typical assembling procedure for the microfluidic device consists of the following steps: (i) a piece of wire about 10 cm long is first introduced through the bottom of the tension holes (4) situated at the edge of the chip, then into the one next to it (2) from the top continuing to the pair of holes (2) and (4) at the other extremity of the chip but in reverse order; at the end of this step, the two wire ends are exiting the side tension holes (4) on the bottom side of the device (path $\Gamma_{1}$ in Figure 2c); (ii) the wire is tensioned by applying a tension force (Figure 2c), then fixed in place (along the path $\Gamma_{2}$) with the help of two conical pins (commercial pipette tips) introduced in the holes (4); (iii) after installing the wires in all 5 channels (by following steps (i) and (ii) for each channel), the two grooves (3) are filled with UV glue (Loctite AA352; Henkel, Düsseldorf, Germany) (7) and cured with the help of a UV lamp (C11924-501 LED controller; Hamamatsu Photonics, Shizuoka, Japan); (iv) the wire is cut between the two tension holes (Figure 2c) and the leftover pieces are extracted from the holes (4) and disposed of; (v) the bottom channels are sealed with a sheet of polymer (AGC, Tokyo, Japan) coated with an adhesive layer (ARclear 93495, 40 μm in thickness; Adhesive Research, Glen Rock, PA, USA); (vi) 10 pieces of tubing (Silastic Laboratory Tubing, 0.76 mm ID, 1.65 mm OD; Thermo Fisher Scientific, Waltham, MA, USA) about 10 cm long each and fitted with commercial fluidic connectors (Qosina, Ronkonkoma, NY, USA) are installed in the two rows of holes (1) and sealed with UV glue for connecting the inlets and outlets of the microfluidic device to the pumping system and flow cytometer, respectively. The microfluidic device weighs about 12.4 g and features 5 incubation/reaction channels with a capacity of 15 μL each. 

### 2.2. Magnetic Field Landscape

At the core of the proposed method for performing immunoassays through magnetic functionalization is the magnetic microwire used in the microfluidic device as a magnetic capture center. The system must allow both the application and removal of an external field to, respectively, magnetize the microwire for capturing the magnetic beads on its surface and complete release of the beads for downstream fluorescence quantification. Consequently, for an efficient and stable functionalization of the wire, the design of the magnetic field applicator has to consider the magnetic properties of both the wire and the magnetic beads used as functional carriers for the assay. 

The physical properties of the FeCuNbSiB alloy used as the magnetic core for the microwire are described elsewhere [24]. One important characteristic of this material is its extremely low magneto-crystalline anisotropy, which means that it is easily magnetized in the presence of an external magnetic field and becomes almost completely demagnetized when the magnetic field is removed (as suggested by both parallel (∥) and perpendicular (⊥) magnetization curves in Figure 2d). Another unique characteristic of this wire is that it is coated with a thin layer of glass (Figure 2b), making it chemically inert, and therefore compatible with biological applications without the need for further coating. Although the magnetization in the parallel direction reaches saturation at very low magnetic fields, the concentration of magnetic poles at the extremity of the wire exclusively makes this configuration not very useful when uniform coating of the entire wire is envisaged. On the contrary, when magnetized in the perpendicular direction, the magnetic poles are uniformly distributed along the wire and, in the absence of any flow, the magnetic particles are actuated with magnetic forces along the radial direction and captured on the surface of the wire. The magnetic field necessary to magnetically saturate the wire in the perpendicular direction is estimated at about 300 kA/m (Figure 2d).

The magnetic particles (amine-modified Dynabeads M270-NH2; Thermo Fisher, Waltham, MA, USA) used here are 3 μm in diameter and are made by incorporating iron oxide nanoparticles in a porous polymer matrix followed by coating with a layer of polymer without charged groups [25] (more details on the surface chemistry and the functionalization are given in Section 2.4). Magnetization measurements confirmed that the particles are superparamagnetic [25] with low hysteresis and saturation fields higher than 400 kA/m (see curve labeled as MB in Figure 2d). 

**Figure 3:**
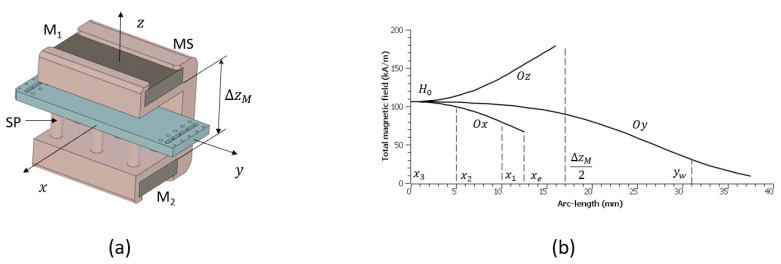
(**a**) Drawing of the magnetic field applicator with the microfluidic device installed in the midplane xOy between the two permanent magnets $M_{1}$ and $M_{2}$; (**b**) numerical magnetostatic simulation of the magnetic field between the two magnet plates. Positions of the 3 wires situated at $x_{1}=10\;mm$, $x_{2}=5\;mm,and$ $x_{3}=0.\;$ $x_{e}$ corresponds to the edge of the microfluidic chip on the Ox direction. Maximum gradient value is $4.8\times 10^{6}\;A/m^{2}$ and corresponds to the edge wire situated at $x_{1}$. Magnetic field at the center of the coordinate system $H_{0}=106\;kA/m$.

For maximum efficiency in terms of magnetic capture and the retention of particles on the surface of the wire, magnetization to saturation is ideally desired for both the magnetic particles and the magnetic microwire. This implies an external magnetic field higher than 500 kA/m, which is difficult to generate in a portable intrument by using relatively small sized permanent magnets. A good compromise has been found by using two BZ0X84 neodymium block-shaped magnets ($M_{1}$ and $M_{2}$) magnetized through thickness (K&J Magnetics, Pipersville, PA, USA) and placed at a distance $\Delta z_{M}=34\;mm$ in a North-South-North-South configuration (Figure 3a). The surface field provided by the magnets is about 120 kA/m with a remanent magnetic induction at a saturation of 1.3 T. In order to hold the magnets together, a support has been designed and fabricated in-house by 3D printing (Form2, Formlabs, Somerville, MA, USA), including an array of supporting pillars (SP) for positioning the microfluidic device with the channels exactly at the midplane between the magnet pair. Accurate numerical magnetostatic simulations made by using an in-house discrete element algorithm [26] suggest a magnetic field with a maximum intensity of $H_{0}=106\;kA/m$ at the center of the midplane between the magnets (the origin of the Oxyz frame in Figure 3a) slowly decreasing towards the edges of the microfluidic device with a slope (gradient) of about $4.8\times 10^{3}\;kA/m^{2}$ in the two side channels (situated at the coordinates $x_{1}=\pm 10\;mm$) and a maximum of about $\nabla H_{max}=5.2\times 10^{3}\;kA/m^{2}$ reached near the long edges of the device ($x_{e}=\pm 12.5\;mm$), as illustrated in Figure 3b. Noticeable variations in the magnetic field are also observed along the Oy axis, but with gradients at the ends of the channels ($y_{w}=\pm 31\;mm$) smaller than $\nabla H_{max}$. For the channels nearer to the center of the magnetic field applicator ($x_{2}=\pm 5\;mm$ and $x_{3}=0$), the field is becoming gradually more uniform with larger variations close to $\nabla H_{max}$ expected at the ends (outlets) of these channels only. When compared to the transverse magnetization curve of the microwire (Figure 2d), the intensity of the magnetic field $H_{0}$ obtained with this pair of magnets is found to be responsible for about 55% of the total saturation magnetization available ($M_{sat}=954\;kA/m$ [24]), that is, $M\left(H_{0}\right)=524\;kA/m$. Analytical magnetostatic models [27] indicate that, in the presence of an external magnetic field of intensity $H_{0}$, the microwire itself contributes to the total magnetic field with an amount $h_{0}=54\;kA/m$ calculated at the surface of the glass shell. The gradient of this field estimated at the same point is about $7\times 10^{6}kA/m^{2}$, which is about three orders of magnitude larger than the maximum gradient $\nabla H_{max}$ generated by the external magnetic field applicator. Consequently, the magnetic carriers (beads) present in the microfluidic channels are actuated magnetically by the magnetic wire only with very low contribution from the magnetic field applicator. In the presence of this magnetic field landscape, the particles are found to be attracted towards the microwire with a magnetic force $F_{m0}=13\;nN$ (asumming the particles reach 80% of their saturation magnetization as suggested by the magnetization curve labeled as “MB” in Figure 2d). Experimental evaluations of particle retention on the magnetic wire in microfluidic flows as large as 100 μL/min demonstrated retention rates of magnetic particles on the glass surface of the wire up to 85%, which was deemed acceptable for the present study.

### 2.3. Pumping System

**Figure 4:**
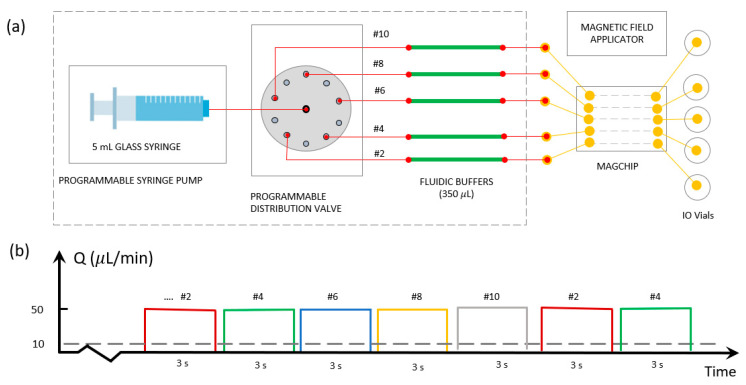
(**a**) Schematics of the microfluidic setup implementing the ICAM-1 immunoassay protocol; (**b**) schematic representation of the profile of the flow across the five channels of the microfluidic device.

The pumping system comprises two parts: (i) a programmable syringe pump (Harvard Apparatus, Holliston, MA, USA) driving a 5 mL glass syringe (Innovative Labor Systems—ILS GmbH, Ilmenau, Germany) connected to a 10-port distribution valve (Advanced Microfluidics SA, EPFL Innovation Park, Ecublens, Switzerland) and then to the microfluidic device (Figure 4a) through commercial tubing (Silastic Laboratory Tubing, 0.76 mm ID, 1.65 mm OD; Thermo Fisher Scientific) and fluidic connectors (Qosina). Five fluidic buffer lines with internal volumes of about 350 μL each are fabricated from larger tubing (~10 cm long; Silastic Laboratory Tubing, 1.8 mm ID, 2.5 mm OD; Thermo Fisher Scientific) and connected between the distribution valve and the microfluidic chip to prevent contamination of the pumping system. Consequently, the fluidic protocol is designed such that the liquids withdrawn by the pump are always contained in these five buffers, and the wash steps are always performed in infusion mode.

During the incubation and wash steps, the pump is run in either infuse or withdrawal mode at a constant pumping rate $Q_{0}=50\;\mu L/min$ while the distribution valve is switching continuously between ports #2, #4, #6, #8, and #10 with a period T=3 s (Figure 4b). For this pumping regime, the liquid is flowing inside each channel for a period of time T at a flow rate of $Q_{0}$ followed by a period of rest (incubation) of 4T which is equivalent to an average flow rate inside each channel of about $Q_{av}=Q_{0}/5$. At the end of the protocol, when the magnetic beads are to be extracted from the microfluidic channel and collected for fluorescence quantification, the magnetic field is removed and the pump is programmed for infusing, withdrawing, and then infusing again through each fluidic line with a higher flow rate $Q_{f}=6Q_{0}$ in order to produce 300 μL of a colloidal suspension of beads at each microfluidic outlet.

### 2.4. Bead Functionalization

Anti-human ICAM-1 antibody (Abcam, Cambridge, UK) was covalently coupled to Dynabeads M270-NH2 particles using glutaraldehyde (Sigma-Aldrich, Oakville, ON, Canada) as a crosslinker. A 100 μL volume of particle suspension ($2\times 10^{9}\;mL^{-1}$) was first washed twice with 1 mL of 1× tris-buffered saline (TBS, pH=7.4; Sigma-Aldrich) using a magnetic rack (Thermo Fisher Scientific) and resuspended in 1 mL of 2% (*v*/*v*) glutaraldehyde in deionized (DI) water. The mixture was incubated at room temperature for 1 h with gentle agitation. Particles were washed three times with DI water. Incubation with capture antibody was performed using a ratio of 97 μg for $2\times 10^{8}$ particles for 1 h at room temperature and overnight at 4 ℃ with gentle agitation. Particles were subsequently quenched by incubation with a 3 M solution of ethanolamine (Sigma-Aldrich) for 1 h, followed by rinsing twice with DI water and blocking with TBST-B (TBS containing 0.1% (*w*/*v*) Tween 20 (Sigma Aldrich) and 0.5% (*w*/*v*) protein blocking reagent) (Bio-Rad, Hercules, CA, USA). After washing, particles were resuspended in 200 μL of TBS and stored at 4 ℃ for up to four weeks until further use. 

### 2.5. ICAM-1 Standard Immunoassay

Standard ICAM-1 ELISA (Abcam, Cambridge, UK) was conducted for calibration purposes in microtiter plates according to the recommendations of the manufacturer. Reference immunoassays involving magnetic particles were performed in tubes. Aliquots comprising $1\times 10^{5}$ particles were incubated with 300 μL of recombinant ICAM-1 (Abcam) in 1× Borate Coupling Buffer (Thermo Fisher) for 1 h at room temperature with gentle agitation. For calibration purposes, ICAM-1 was serially diluted to provide absolute quantities ranging from 3 pg to 3 ng per tube. Upon removal of the supernatant, particles were washed three times using TBST and incubated with 1.5 μg of biotinylated detection antibody (Abcam) in 300 μL of TBST for 1 h at room temperature using gentle agitation. Removal of the supernatant and washing of the particles was followed by incubation with 300 μL of Cy5-conjugated streptavidin (diluted 1:50 (*v*/*v*) in TBST; Sigma-Aldrich) for 20 min at room temperature using gentle agitation. Finally, particles were washed and resuspended in 100 μL of TBST for fluorescence imaging and flow cytometry measurements. For the measurements of ICAM-1 in real human samples, plasma was collected from the blood samples obtained from healthy volunteers using standard density gradient centrifugation (Ficoll-Paque from Sigma-Aldrich). The as-obtained plasma was subsequently diluted at a ratio of 1:10 and 1:20 in PBS to perform the immunoassay.

### 2.6. Flow Cytometry Fluorescence Quantification

**Figure 5:**
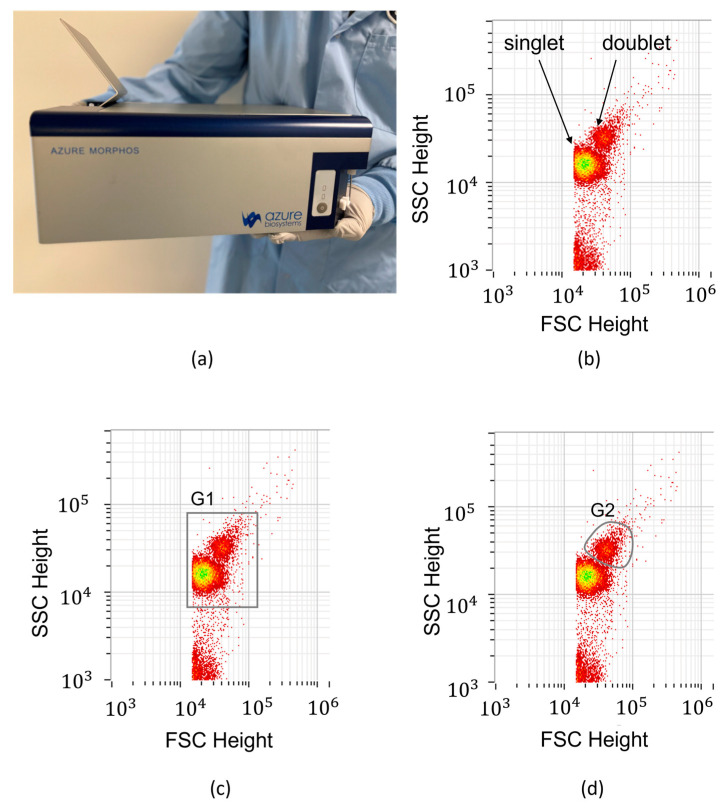
Photograph of the AZ-150 Personal and Portable Cytometer produced by Azure Biosystems Inc. (**a**) and screen captures of the FSC-SSC diagrams used for gating and fluorescence quantification of the beads (**b**–**d**).

Following the microfluidic immunomagnetic assay, 300 μL of colloidal suspensions of magnetic beads were extracted from each of the five microfluidic channels. These samples were then quantified by using a portable flow cytometer (AZ-150 Personal and Portable Cytometer, Azure Biosystems, Montreal, QC, Canada). Although automation of the transfer of the samples to the fluorescence measurement instrument is possible, the output samples were manually collected and transferred successively to the flow cytometer inlet by using Eppendorf tubes (Figure 5a). The quantification was performed for the Cy5 fluorophore by monitoring the average fluorescence per event (bead) on the channel FL-4 of this instrument. On an FSC-SSC diagram, a typical signature of our samples (Figure 5b) consists of some inherent noise cloud located at the bottom of the diagram and two central disk-shaped clouds related to our magnetic particles. The larger (and central) one was found to be associated with singlet-state particles, while the smaller one at the top-right corner corresponds to larger aggregates, formed of particles clogged together. By considering these aggregates in a first approximation as doublet configurations, the quantification is then performed as follows. We first read the total number of events $N_{tot}$ and the average Cy5 fluorescence value $I_{FL4}$ in a gate G1 containing both singlet and doublet types of events (Figure 5c). Then, we isolated the doublet events in a (polygonal) gate G2 as shown in Figure 5d and evaluated the number of events $N_{2}$ in this gate. With these three quantities, the average total fluorescence intensity per bead is given by

(1) $$I_{FL4}^{corr}=\frac{N_{tot}I_{FL4}}{N_{tot}+N_{2}}$$

Then, the average fluorescence per bead relative to the control beads (stock particles) is obtained by subtracting the intrinsic fluorescence intensity $I_{FL4}^{stock}$ of the stock beads from the absolute value in Equation (1) as follows:

(2) $$I_{FL4}=I_{FL4}^{corr}-I_{FL4}^{stock}$$

This quantity is measured in ERF (Equivalent Reference Fluorophore number [28]) and is considered as being proportional to the average number of proteins captured on the surface of the beads. By performing several microfluidic assays with input samples of well-known concentrations of recombinant ICAM-1, we can obtain accurate calibration curves for the system and use them for further quantification of real samples.

### 2.7. Microfluidic Assay and Fluidic Protocol

The microfluidic protocol for conducting a complete ICAM-1 immunoassay with the microfluidic chip is presented in Table 1. First, the syringe is manually filled by disconnecting it from the distribution valve and withdrawing 5 mL of wash buffer. Five vials are installed at the device outlets (labeled as “IO vials” in Figure 4a) and the syringe is reconnected to allow infusion of 1 mL of wash buffer along each fluidic line/channel (step 1) which was found enough to completely fill each of the five fluidic lines. Before introducing the magnetic beads in the microfluidic channels, we ensure that the beads do not diffuse and spread along the connection tubes into the pumping system. To achieve this, an air bubble is artificially created on each fluidic line by disconnecting the outlets and withdrawing 50 μL of air into their respective connecting tubing (step 2). The most important step in the assay protocol is the magnetic functionalization of the wire inside the microfluidic channel (step #3, Table 1): 15 μL of a colloidal suspension containing about $10^{6}$ magnetic beads functionalized with anti-ICAM-1 antibody are withdrawn successively into each channel through the microfluidic device outlets. The chip is then installed in the magnetic field applicator to apply the magnetic field and transversally magnetize the microwire. At this stage, the magnetic beads in the solution are actuated by the magnetic field gradient generated by the wire, causing them to move toward it and form a coating (ideally a monolayer) on its surface. Subsequent assay steps are repetitive, alternating between incubations with the sample (step #5, Table 1), the detection antibody (step #7), and the fluorescence labeling (step #9), as well as the wash steps in between different incubations (steps #4, #6, #8, and #10). At the end, after the fluorescent labeling of the beads, the magnetic field is removed and a high flow rate washing of the channel is performed (step #11) in order to extract the beads and prepare them for flow cytometry fluorescence quantification. As indicated in Table 1, the total microfluidic assay time takes approximately 2 h and 31 min, including the time necessary for performing the fluorescence measurements. 

## 3. Results and Discussion 

**Figure 6:**
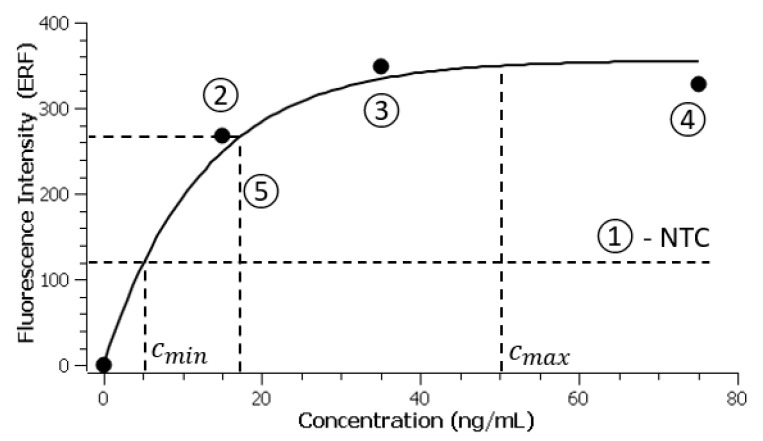
Quantification human blood plasma sample on channel #5 with negative control on channel #1 and three other recombinant solutions with known concentration values on channels #2, #3, and #4.

In previous calibration assay experiments (following the procedure described in Section 2.5), we have prepared solutions spiked with concentrations of recombinant ICAM-1 ranging from 0 ng/mL (negative control) to 75 ng/mL. These experiments were then used to identify the coefficients A and B in the analytical model

(3) $$I\left(C\right)=A\left(1-e^{-BC}\right)$$

through interpolations in the least square sense (using the SciDavis analysis software version 2.7 [29]) and by excluding the zero-concentration data, as suggested elsewhere [30]. The coefficient A generally varies from one experiment to the other and corresponds to the intensity of the fluorescence signal at very high concentrations (saturation), while B=0.069±0.006 mL/ng is more consistent and related to the sensitivity of the immunoassay itself. This suggests a testing strategy in which the coefficient B is considered as well known and precisely identified from previous experiments, while the other coefficient A is always identified along with the sample in the same run as a “positive” reference. The complete strategy for a test assay was therefore as follows: (i) at least one channel is used as a negative control where non-spiked wash solution will be loaded at the sample inlet; (ii) at least two channels are exposed to solutions with relatively high concentrations of recombinant ICAM-1 to provide the intensity of the fluorescence signal at saturation (coefficient A); (iii) use the remaining channels for quantifying real samples. Figure 6 shows the experimental results for a test where we employ channel 1 as a negative control, channels 2, 3, and 4 as high-concentration recombinant controls for identification of the parameter A while the remaining channel 5 is used for quantifying a real human plasma sample with a known concentration of ICAM-1 (251 ng/mL, as evaluated through standard ELISA). Upon completion of the microfluidic assay, the five samples are subjected to Cy5 fluorescence measurements using the procedure described in Section 2.6. As a reference, three samples containing bare stock particles are measured at the beginning of each experiment, and the average of these measurements is used as $I_{FL4}^{stock}$ in Equation (2), that is, the fluorescence level corresponding to the zero point on the calibration curve. Using this value, the fluorescence level at the three concentrations on channels 2, 3, and 4 are evaluated with Equation (2) and used in conjunction with the (0, 0) point to identify the coefficient A through interpolation in the least square sense with the analytical model (3). The value we find by following this strategy is A=355.7±16.3 ERF and corresponds to a maximum capacity (saturation point) of about 356 fluorophores per magnetic particle. With the two parameters of our model A and B identified, we can now evaluate the concentration of the sample loaded at the channel 5 by solving the equation $I\left(C_{x}\right)=I_{x}$, where $I_{x}=267\;ERF$ is the intensity of the fluorescence measured for the unknown plasma sample:

(4) $$C_{x}=\frac{k}{BD}ln\frac{A}{A-I_{x}}$$

For a dilution factor D=0.1 and a ratio k=1.6, between the molecular mass of the ICAM-1 protein and the recombinant, we obtain $C_{x}=241\;ng/mL$, which is very close to the known value of 251 ng/mL previously determined using standard ELISA. Standard quadrature error analysis [31] applied to our measurements suggests an absolute error of 43 ng/mL propagating through the coefficients A and B in Equation (4), which represents about 17% of the measured value in terms of relative error. However, the most significant contributing factor to this error originates from the parameter B, which accounts for almost 10%. Since this coefficient is identified through separate calibration experiments, the overall measurement error could, in principle, be reduced and limited to the error in the identification of the coefficient A by envisaging extensive calibration assays and more precise identification of the B parameter. The value corresponding to the negative control (dashed horizontal line in Figure 6) is used as a limit of detection for our assay and corresponds to $c_{min}≅5\;ng/mL\;(any$ measurement smaller than this value is considered as being out of range).

We essentially attribute the most part of the imprecision in the ICAM-1 measurements to analytical factors, namely particle functionalization and antibody stability. While glutaraldehyde coupling constitutes a plausible strategy for immobilizing antibodies [32] that we have successfully used for conducting immunoassays on polymer substrates [33], it also has drawbacks: glutaraldehyde reacts with a variety of nucleophilic groups, and thus can result in random modification and orientation of proteins [34,35,36], altering their activity, structure, and function in unpredictable ways. Moreover, the fact that only a relatively small number of proteins are accommodated (and detected) on a single (and comparatively large) particle suggests that the immobilization protocol is somehow inefficient. For this reason, even minor differences in protein density can have a pronounced effect on variability between measurements relative to one another since each fluorophore counts. The use of alternative coupling chemistries (e.g., involving carbodiimide) can potentially achieve more controlled and specific immobilization with better yield and fewer side effects. We also observed that storage of particle aliquots reduces capture efficiency for ICAM-1 over time. The addition of stabilizing agents (such as trehalose, glycerol, or proline) needs to be envisaged for mitigating time-dependent variation in the case of prolonged delays between particle functionalization and use. It should be noted, however, that even high coefficients of variation can provide statistically significant outcomes for quantitative protein measurements, provided that an adequate parameter space is considered for both experimentation and analysis [37].

Currently, the assay duration is relatively long, primarily due to the limitation of using a syringe-based pumping system and a distribution valve to direct the flow to different channels in the microfluidic device. A significant reduction in assay time (at least five-fold) could be achieved by running the assay in parallel, where flow is distributed simultaneously to all five channels, rather than serially through the distribution valve. This can be accomplished by either using individual syringes for each channel or employing a parallel pressure-driven pumping system instead of the syringe pump.

The magnetic field applicator is static, and the beads remain immobilized on the microwire throughout the assay. The downside of this approach is that mainly the outer surface of the functionalized beads participates in the capture reaction, as the area facing the wire is more difficult to access. Consequently, the total capacity of the beads is reduced, and it is expected that the saturation point (coefficient A) would roughly double if the beads were fully exposed to the sample flow. A more sophisticated magnetic field applicator could dynamically apply and remove the magnetic field during the incubation steps, allowing the beads to periodically be released and recaptured, ensuring full exposure to the sample flow. Additionally, during the final release step, when the beads are prepared for fluorescence quantification, incorporating additional mixing steps could help reduce aggregation and decrease the size of the secondary cloud G2 (Figure 5d). Despite this expected reduction in terms of dynamic range when using a static magnetic field applicator, the microfluidic approach has the capacity to outperform the classical benchtop approach through increased accuracy (smaller error bars) enabled mainly by full automation as well as low costs due to (i) reductions in the total volume of the reaction chambers and reagents, (ii) using a smaller number of beads per reaction chamber, (iii) using soft magnetic microwires as magnetic capture centers and (iv) functionalizing the microfluidic chip “on-the-fly” through a magnetic functionalization strategy.

Although the microfluidic device presented here contains only five channels, designs and pumping systems capable of accommodating more channels per run are also feasible (10 channels with the current setup, or even more if the distribution valve is upgraded as well). This would allow for the simultaneous processing of multiple samples and the inclusion of more sophisticated concentration gradients, leading to more precise identification of the model parameters. The measurement error could also be substantially reduced by both conducting more experiments to refine identification of the parameter B and by fine-tuning the optimal dilution factor.

## 4. Conclusions

A complete magnetic immunoassay for the quantification of proteins in biological samples has been developed. At the core of the method is a microfluidic device featuring five reaction chambers (channels), which is activated by magnetically assembling functionalized magnetic beads around soft magnetic capture centers in the form of long, glass-covered soft magnetic wires. The microfluidic device is driven by a pumping system compatible with syringe-based flow cytometry analysis systems, enabling straightforward fluidic connectivity. A complete functionalization workflow, along with an automated fluidic protocol, demonstrates great potential for direct fluorescence quantification of proteins in human plasma samples by combining microfluidics with miniature flow cytometry instruments. 

## Figures and Tables

**Table 1:** Microfluidic protocol.

#	Step	Description	Time (min)
1	Initial filling	The syringe is manually filled with wash buffer after disconnecting it from the distribution valve. Then, the syringe is reconnected and the pump set to infuse wash buffer until it exits through each of the output outlets of the microfluidic device.	5
2	Air priming	An air bubble is formed at the outlets by withdrawing 50 μL of air on each fluidic line.	3
3	Magnetic functionalization	A volume of 15 μL$\;\mathrm{of}\;a\;\mathrm{colloidal}\;\mathrm{suspension}\;\mathrm{containing}\;\mathrm{about}\;10^{6}$ magnetic beads functionalized against ICAM-1 are introduced manually in each microfluidic channel. After this operation is complete, the chip is installed and secured in the magnetic field applicator.	3
4	Wash #1	Infuse 300 μL of wash buffer per channel	10
5	Sample incubation	Connect the outlet of the microfluidic device to the sample tubes and perform the incubation with the sample (withdraw and then infuse 300 μL per channel)	30
6	Wash #2	Infuse 300 μL of wash buffer per channel	10
7	Detection antibody	Connect the outlet of the microfluidic device to the tubes containing the detection antibody and perform the incubation (withdraw and then infuse 300 μL per channel)	30
8	Wash #3	Infuse 300 μL of wash buffer per channel	10
9	Fluorescence labeling	Connect the outlet of the microfluidic device to the tubes containing the fluorescently (Cy5) tagged streptavidin and perform the incubation (withdraw and then infuse 150 μL per channel)	15
10	Wash #4	Infuse 300 μL of wash buffer per channel	10
11	Bead extraction	Remove the microfluidic device from the magnetic field applicator and perform the bead removal protocol by infusing, withdrawing and then infusing again 300 μL of wash buffer into 5 clean Eppendorf tubes.	15
12	Quantification	Perform fluorescence quantification of the samples by using the flow cytometer.	10
Total microfluidic assay time (min)			151

## Data Availability

The original contributions presented in the study are included in the article, further inquiries can be directed to the corresponding author.
